# Transcriptomic signatures of host immune responses in aphthous ulcers, the earliest lesions of Crohn's disease, suggest that bacterial uptake, rather than global dysbiosis, is the initiating factor

**DOI:** 10.1111/imcb.70031

**Published:** 2025-05-19

**Authors:** Phillip J Whiley, Ojas VA Dixit, Mukta Das Gupta, Hardip Patel, Guoyan Zhao, Susan J Connor, Kim M Summers, David A Hume, Paul Pavli, Claire L O'Brien

**Affiliations:** ^1^ School of Medicine and Psychology Australian National University Canberra ACT Australia; ^2^ Gastroenterology and Hepatology Unit Canberra Hospital Canberra ACT Australia; ^3^ John Curtin School of Medical Research College of Health and Medicine, Australian National University Canberra ACT Australia; ^4^ Department of Pathology & Immunology Washington University School of Medicine St. Louis MO USA; ^5^ South Western Sydney Clinical School University of New South Wales Sydney NSW Australia; ^6^ Department of Gastroenterology Liverpool Hospital Sydney NSW Australia; ^7^ Ingham Institute Sydney NSW Australia; ^8^ Mater Research Institute—University of Queensland Translational Research Institute Brisbane QLD Australia; ^9^ Faculty of Science and Technology, University of Canberra Canberra ACT Australia

**Keywords:** bowel, epithelia, macrophage, pathology, plasma cell

## Abstract

Crohn's disease is a chronic, transmural inflammatory disease of the human gut. Changes in the fecal microbial composition and dysbiosis are consistent features in studies of Crohn's disease patients, but whether dysbiosis is a cause or consequence of inflammation remains unresolved. Genetic susceptibility plays a role in the development of Crohn's disease and has been linked to genes involved in recognition of intestinal bacteria by the mononuclear phagocyte system. The earliest visible lesions in Crohn's disease are aphthous ulcers, overlying Peyer's patches and lymphoid follicles. To identify mechanisms underlying the earliest stages of disease we compared gene expression in aphthous ulcers, Peyer's patches, inflamed and endoscopically normal mucosa from patients and controls using total RNA‐seq. The resulting data were subjected to network analysis to identify coregulated gene expression signatures of cell types and processes. These results were compared to single‐cell RNA‐seq analysis of intestinal macrophages in normal and diseased mucosa. The analysis of aphthous ulcers revealed signatures of epithelial stress and antimicrobial defense, plasma cell activation and immunoglobulin production, monocyte recruitment, inflammatory gene expression and induction of interferon‐γ. These signatures were not present in the normal appearing mucosa adjacent to aphthous ulcers, which were similar to healthy control mucosa. Given the role of Peyer's patches and lymphoid follicles in sampling the luminal contents, these findings suggest the initial lesion in Crohn's disease arises from the uptake of bacteria and the activation of multiple host defense pathways rather than the breakdown of epithelial barrier integrity and widespread bacterial translocation.

## Graphical abstract

To identify mechanisms underlying the earliest stages of disease initiation, we compared gene expression in aphthous ulcers, Peyer's patches, inflamed and endoscopically normal mucosa from patients and controls using total RNA‐seq. The signatures obtained suggest epithelial stress, plasma cell activation, monocyte activation and interferon‐γ signaling are driving the dysregulated response. We conclude that the initial lesion in Crohn's disease arises from an invasive bacterial challenge leading to intense activation of multiple host defense pathways rather than the breakdown of epithelial barrier integrity and widespread bacterial translocation.

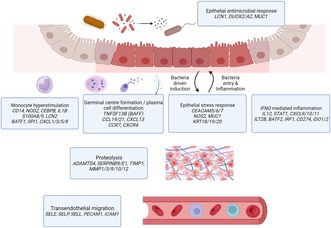

## INTRODUCTION

Crohn's disease is a debilitating chronic inflammatory condition affecting the gastrointestinal tract. Changes in the microbial composition and a reduction in species diversity (dysbiosis) are consistent features in studies of Crohn's disease patients. It is postulated that these changes affect epithelial barrier integrity, permitting translocation of bacteria and their products and/or compromise the host inflammatory response.^1^

Genetic susceptibility also plays a role in the development of CD: based upon results from genome‐wide association studies (GWAS), it is widely believed that heritable disease susceptibility is linked to hyper‐responsiveness of cells of the monocyte–macrophage lineage to gut microbiota.^2^ Very early onset forms of IBD inherited in a Mendelian fashion are commonly due to mutations in monocyte–macrophage‐expressed genes, and Crohn's disease susceptibility loci identified by GWAS are strongly enriched for regulatory elements associated with monocyte to macrophage differentiation and/or activation.^3^

In an effort to identify inflammatory mechanisms and therapeutic targets, the past 10 years have seen numerous published transcriptomic analyses using either total RNA sequencing (RNA‐seq) or single‐cell RNA sequencing (scRNA‐seq), comparing affected and unaffected areas of patient GI tract.^3^, ^4^, ^5^, ^6^, ^7^, ^8^ Not surprisingly, many immune response‐related genes are overexpressed in the inflamed bowel.

The potential weakness of these studies is the focus on established lesions in inflamed mucosa. A seminal study of postoperative recurrence in Crohn's disease patients demonstrated that aphthous ulcers are the earliest detectable lesions.^9^ These lesions overlie the follicle associated epithelium (FAE) of the small bowel (Peyer's patches) and large bowel (lymphoid follicles) and were detected in 70% of patients with Crohn's disease.^9^ There is a predictable sequence from aphthous ulcers to typical ileitis.^10^ Radiological and endoscopic studies^11^ showed that about 30% of patients with an “incidental” finding of isolated terminal ileal aphthous ulceration subsequently develop Crohn's disease.

Peyer's patches and colonic lymphoid follicles are secondary lymphoid structures that facilitate the interaction between gut antigens and antigen‐specific lymphocytes leading to the induction of intestinal immunoglobulin and other responses.^12^ Luminal antigens are delivered by specialized microfold (M) epithelial cells within follicle‐associated epithelium to underlying antigen‐presenting cells to initiate immune responses. M cells also provide a route of entry for various pathogens, such as *Yersinia pseudo‐tuberculosis*, *Salmonella typhimurium* and *Shigella flexneri*.^13^ Adherent‐invasive *Escherichia coli* adhere to follicle‐associated epithelium and were more commonly isolated from mucosal biopsies of patients with Crohn's disease than controls.^14^ Increased uptake of nonpathogenic *E. coli* has also been demonstrated in longstanding Crohn's disease but not ulcerative colitis.^15^ These studies led to speculation that the microbial triggers of Crohn's disease gain access to the intestinal lamina propria via Peyer's patches.^16^

To identify mechanisms underlying the earliest phases of CD, we focused on aphthous ulceration in a unique group of previously diagnosed Crohn's disease patients who were not on treatment. We compared gene expression and microbial profiles of aphthous ulcers and adjacent mucosa and Peyer's patches and adjacent mucosa from controls. To compare with established disease and to highlight responses that are specific to inflamed Peyer's patches, we also profiled involved and adjacent mucosa and mesenteric lymph nodes from patients with active disease. In parallel, we characterized the mucosa‐associated virome and microbiome of the earliest identifiable lesion in Crohn's disease to identify candidate triggers that could be associated with the initiation of disease. We conclude that aphthous ulcers are associated with evidence of microbial uptake, an epithelial stress response, B‐cell activation and antibody production and activation of recruited monocytes to produce inflammatory cytokines. These changes were not seen in the adjacent normal appearing mucosa. Network analysis of the data highlights informative co‐expression signatures of different mucosal cell populations and is discussed in comparison to recent large scRNA‐seq data sets from normal and inflamed human intestine.^17^

## RESULTS

### Network analysis

Total RNA‐seq data were generated from a total of 48 biopsies, including aphthous ulcers, control Peyer's patches, normal‐appearing mucosa from Crohn's disease patients and healthy controls, inflamed and normal‐appearing mucosa and lymph nodes from Crohn's disease patients with active disease undergoing intestinal resection. On average, we obtained 36 million reads per sample of which 87% were retained for downstream processing, and 93% of these mapped to the human genome. Gene expression was quantified as described in the Methods. The complete set of quantified expression data is provided in Supplementary table 1. Aside from the differences in location (Peyer's patches, mucosa, lymph node), individual biopsies from the same location also differ in cellular composition and activation status. Network analysis exploits this diversity to enable extraction of sets of correlated transcripts that define cell types, subtissue locations and/or cellular processes. This approach has been used previously to identify cellular signatures in very large data sets including those derived from human tumours.^18^

**Figure 1:**
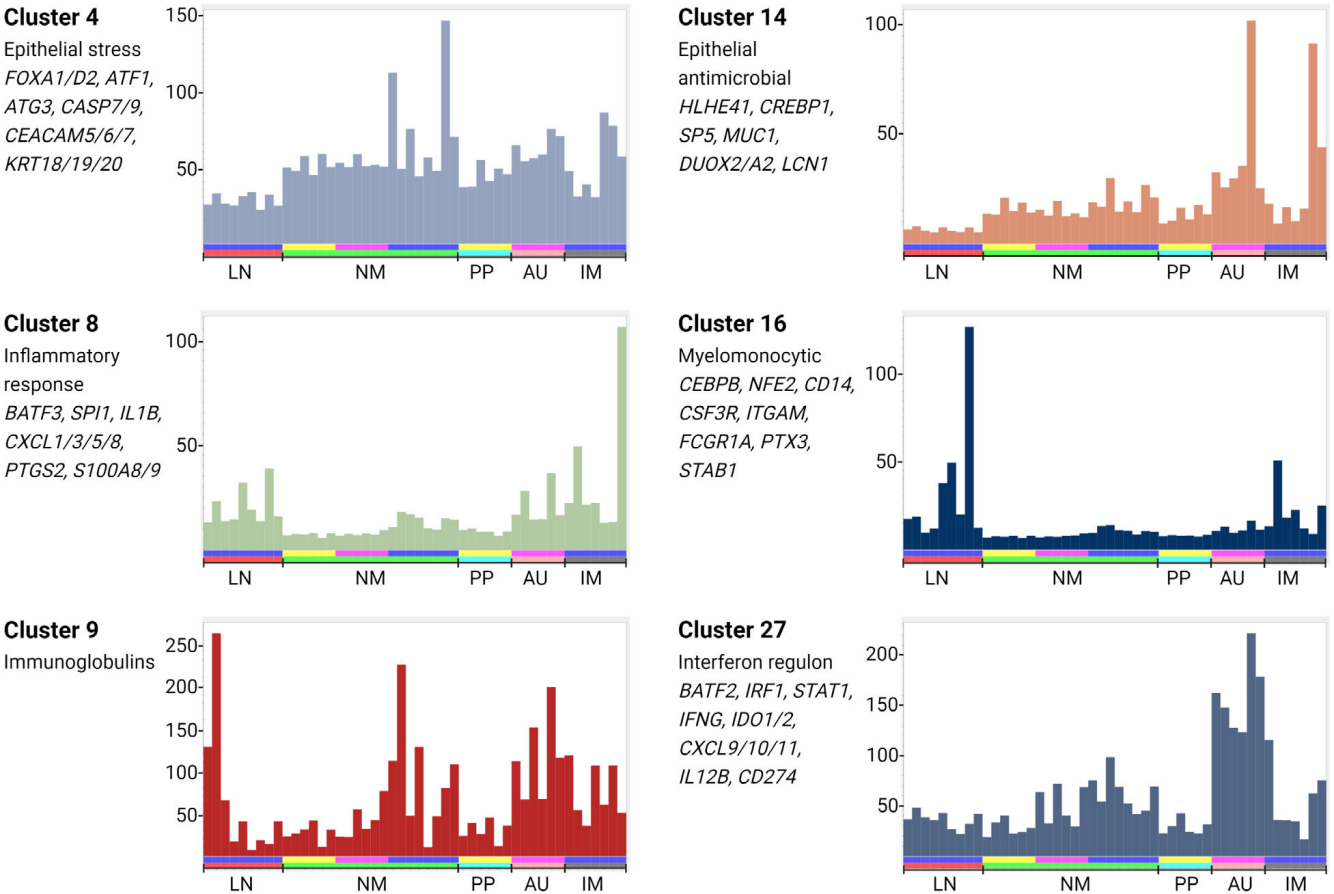
Co‐expression clusters identified from gene‐centered network analysis. Total RNA‐seq data were generated from 48 biopsies, including aphthous ulcers (AU), control Peyer's patches (PP), normal‐appearing mucosa from Crohn's disease patients and healthy controls (NM), inflamed mucosa/active disease (IM) and lymph nodes (LN) from Crohn's disease patients with active disease. A gene‐centered network was generated using BioLayout as described in the Methods. The full list of clusters is provided in Supplementary table 2. Histograms in this figure show the average expression profile of the set of transcripts in clusters of specific interest, an indication of the shared pattern of expression. *y‐*axis: average expression (transcripts per million, TPM). *x*‐axis: samples. Upper bar: blue—Crohn's disease patients with active disease, pink—aphthous ulcer Crohn's disease patients, yellow—controls. Lower bar: red—lymph node, green—normal mucosa, turquoise—Peyer's patch, salmon—aphthous ulcer, gray—involved mucosa.

A gene‐centered network (GCN) was generated at a threshold Pearson correlation of *r* = 0.75 and a Markov clustering (MCL) inflation value (defining granularity) of 2.0. As in previous studies, the threshold was chosen empirically to maximize the number of nodes and minimize the number of edges. Supplementary table 2a, b contains the lists of genes and the average expression profiles of clusters of interest and annotation of the clusters highlighting the power of the analysis to extract informative co‐expression signatures of different cell types and processes. Among the clusters, a subset showed a pattern of shared expression corresponding to an inflammatory state. **Clusters 4, 8, 9, 14** and **27** distinguished aphthous ulcers from Peyer's patches; of these, gene expression in **Clusters 8** and **14** were also increased in inflamed mucosa or lymph nodes from Crohn's disease patients (Figure 1). Six clusters were assessed for enrichment with known and candidate susceptibility genes.^19^ Taken in combination, **Clusters 4, 8, 9, 14, 16** and **27** contained 30 of 231 known IBD susceptibility genes and 28 of 153 monocyte candidate genes, respectively. **Cluster 20** includes markers of several distinct cell types in intestinal crypts. **Cluster 21** contains markers consistent with lamina propria macrophages, whereas **Cluster 25** contains markers of submucosal macrophages, and Cluster **16** contains the monocytic transcription factor, *CD14*.

**Figure 2:**
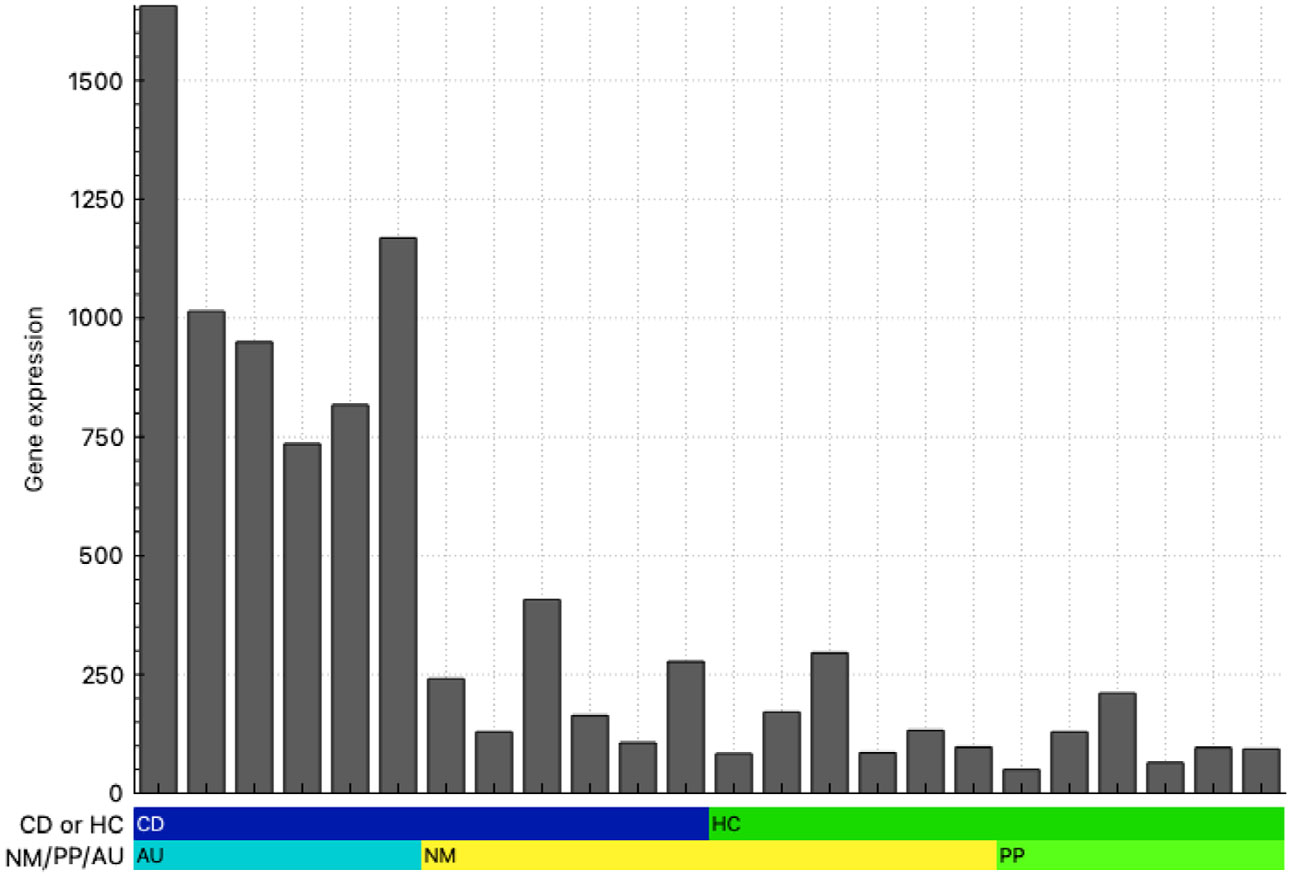
Epithelial cell‐specific gene network analysis. Total RNA‐seq data were generated from 24 biopsies, including aphthous ulcers (AU), Peyer's patches (PP) and normal‐appearing mucosa from Crohn's disease patients and healthy controls (NM). A gene‐centered network was generated using Biolayout as described in the Methods. Histograms show the average expression profiles of the set of epithelial stress/bacterial response gene transcripts of interest (Table 1). *y‐*axis: average expression (transcripts per million, TPM). *x‐*axis: samples. Upper bar: blue—Crohn's disease patients, green—healthy controls. Lower bar: turquoise—aphthous ulcer samples from six Crohn's disease patients, yellow—normal‐appearing mucosa from the six corresponding Crohn's disease patients in sequence and normal mucosa from six healthy controls and lime green—Peyer's patches from the corresponding six healthy controls in sequence.

To determine whether or not epithelial injury was evident in mucosa adjacent to aphthous ulcers, we examined the expression of 23 epithelial specific genes (biogps.org) (Table 1), which shows a list of genes, excluding immunoglobulin genes, that have been curated into broad functional classes, and are upregulated in aphthous ulcers compared to adjacent mucosa (Table 1) (https://biogps.org), and compared aphthous ulcers, the adjacent normal appearing mucosa in the same subject, Peyer's patches and the adjacent mucosa in the same subject (Figure 2). There was no evidence for epithelial injury in the mucosa adjacent to Peyer's patches or aphthous ulcers to suggest a more global insult.

**Table 1:** Functional classification of transcripts upregulated in aphthous ulcers compared to Peyer's patches (the full list of regulated transcripts is shown in Supplementary table 3a).

Biological function	Genes of interest
Epithelial stress/bacterial response	*AGR2, ALDH1A2, CD55, CD24, CEACAM5/6/7, CHI3L1, DUOX2/A2, TFF1/2, PRRX1, CLCA4, HAPLN3, PLEK, FFAR4, DAPP1, IGFBP5, PARM1, SLC2A1/2A6/6A14, SLC7A5/7A11, HCAR3, CEMIP, TFPI2, FOLH1/1B, REG1A/1B, MUC1/4/12, PLAUR, S100P, NOS2, SPINK4, SOD2*
Chemokines and receptors	*CXCL1/2/3/5/6/8/9/13* *CCL2/3/4/19/20/22* *CXCR1/2/4/5, CCR7*
Cytokines	IL1B, IL6, IL12B*, IL23A*, TNF, LTB, CSF3, OSM, INHBA, EBI3, IL1RN, FGF7, HGF, ADM
Interferon response	*IFNG, STAT1, BATF2, LITAF*, *IFITM2/3, IFI6, GPR183, RSAD2, SOCS1/3, CLEC4E, CTLA4, CXCL10/11, GZMB, IDO1, KYNU, MNDA, OASL, TDO2*
Cell surface receptors and myeloid markers	*SPI1, CEBPB*, *CSF2RB, CSF3R, IL3RA, CD14, CD22, CD72, CD80, CD83, CD274, C5AR1 FPR1/2, LILRB3, CD300E, CYBB, NCF1/2, TREM1, TMEM163, ITGAX, IL4I1, OSMR, CLEC4A, FCGR1A/2B/3A/3B, LRRK2, FCER2, FCRL3, SLC11A1, LILRA5/B1/B2/B4, TLR2/4/8/10*
B cells and immunoglobulin	*PAX5, CD19, CD79A/B, BLK, BTK, IGHD, IGHM, IGLC2, IGHG1/2/3/4, IGHA1*
Proteolysis	*ADAMTS4, CPM, MMP1/3/9/10/12, CTSE, TIMP1, PLAU, SERPINB9/E1, SLPI*
Inflammatory markers and regulators	*SAA1/2, S100A8/9/11, TNFAIP2/8, FCN1, LCN2, PTGS2, PTGDS, PTGES, PTAFR, SPP1, DUSP4, FGR, IER3*
Vascular adhesion	*SELE, SELL, SELP, S1PR3/4, MCAM, MADCAM1, ICAM1, VWF, PECAM1*
Epithelial cell‐specific	*AGR2, CD24, CEACAM5/6/7, CLCA4, DUOX2/A2, FFAR4, FOLH1/1B, MUC1/4/12, NOS2, REG1A/1B, SLC6A14, SLC7A11, SOD2, SPINK4, TFF1/2*

### Differential gene expression

Network analysis groups transcripts that share a pattern of expression, but the basal level of expression and fold differences vary for transcripts within a cluster. Furthermore, depending upon the correlation threshold, some additional differentially regulated genes will form smaller clusters based upon idiosyncratic differences between individual biopsies. Supplementary table 3a shows the results of a conventional differential gene expression analysis, comparing aphthous ulcers and Peyer's patches. Excluding transcripts that were not detected at > 5 transcripts per million (TPM) on average in aphthous ulcers biopsies, there were 1026 transcripts increased significantly in aphthous ulcers *versus* Peyer's patches. We also compared gene expression in paired samples of aphthous ulcers and the adjacent normal appearing mucosa. These are broadly annotated in a ranked list in Supplementary table 3b. Table 1 shows a curated set of genes that were upregulated in aphthous ulcers excluding immunoglobulin variable genes and classified into broad functional classes based upon the known gene function. IL23 (subunits encoded by *IL12B* and *IL23A*), which is strongly implicated in IBD pathology,^20^ fell below the 5 TPM detection threshold, but both transcripts were increased > 5‐fold in aphthous ulcers *versus* Peyer's patches.

**Figure 3:**
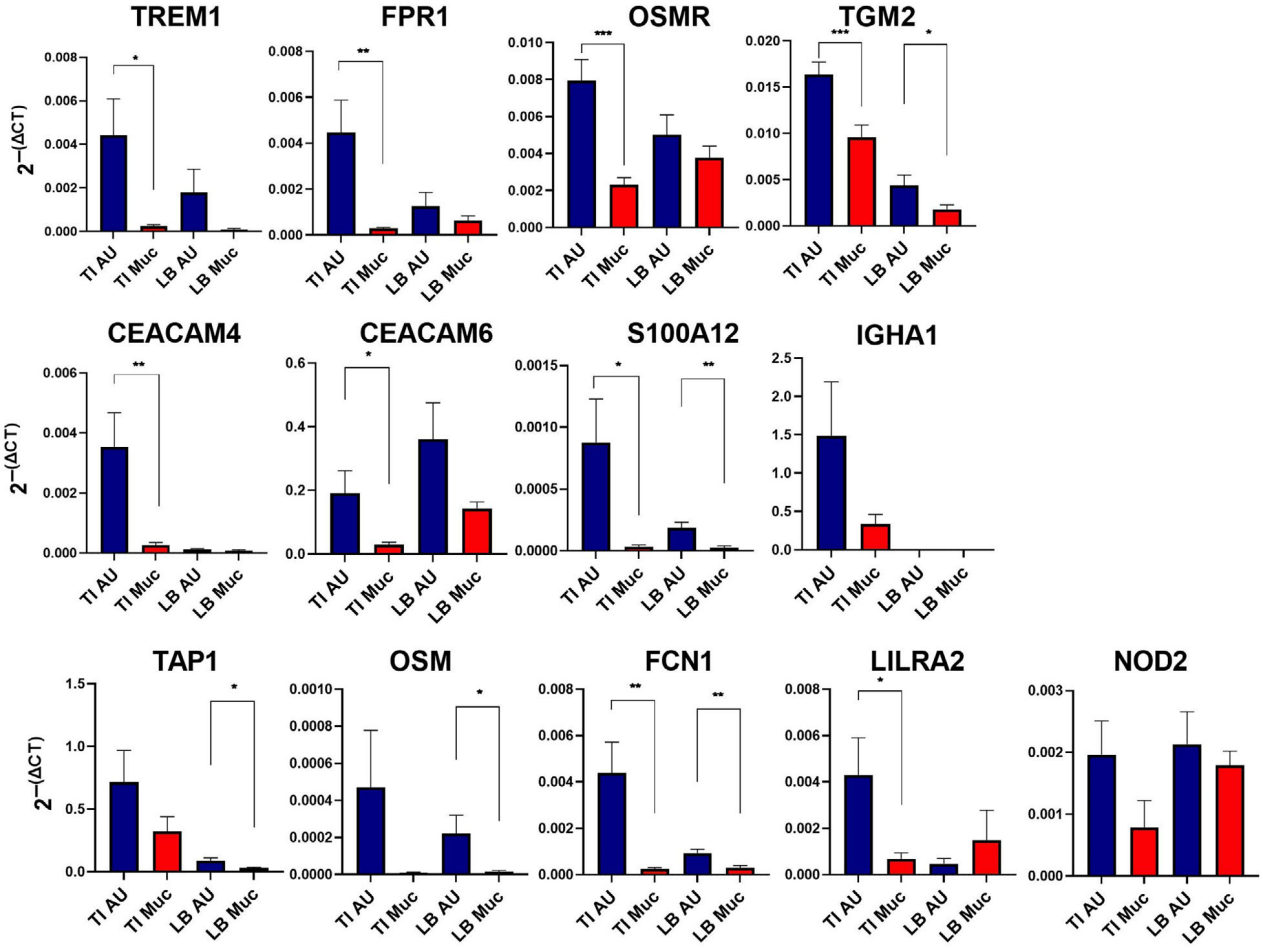
Expression of selected transcripts in aphthous ulcers and adjacent mucosa. mRNA was isolated from terminal ileum aphthous ulcers (TI AU), adjacent terminal ileum normal appearing mucosa (TI Muc), large bowel aphthous ulcers (LB AU) and large bowel normal appearing mucosa (LB Muc), and expression of the indicated transcripts was determined by qRT‐PCR as described in the Methods. *P < 0.05, **P < 0.01, ***P < 0.001 for pairwise comparison of affected *versus* unaffected tissue.

To validate the patterns of altered gene expression seen in the RNA‐seq analysis, we performed qRT‐PCR analysis on 13 transcripts selected from Table 1, comparing aphthous ulcers and adjacent mucosa in the terminal ileum and colon obtained from a separate cohort of Crohn's disease patients. Transcripts were chosen to sample novel genes of interest across a range of biological functions and distinct clusters, including myeloid markers (*TREM1, FPR1, OSMR, LILRA2, NOD2*), inflammatory diagnostic markers (*S100A12, FCN1*), an antibody (*IGHA1*), a cytokine (*OSM*), antigen processing (*TAP1*) and epithelial response (*CEACAM4/6*). *TGM2* was recently identified as a novel marker of epithelial responses in experimental and human colitis.^21^ All 13 transcripts were significantly upregulated in aphthous ulcers in the terminal ileum. The pattern was more variable in the colon, where *IGHA* was not detected and *LILRA2* and *NOD2* were unaffected (Figure 3). To extend the analysis, we compared the expression of the same set of transcripts in actively inflamed tissue to either uninflamed mucosa adjacent to aphthous ulcers or mucosa from non‐IBD controls in the terminal ileum (Supplementary figure 1) or in the colon (Supplementary figure 2). All transcripts except *TAP1* distinguished active inflammatory mucosa from inactive and/or control mucosa in the terminal ileum. However, in the colon, the association with involved mucosa was less evident. Whereas 9/13 transcripts (excluding *TAP1, TGM2, IGHA1* and *CEACAM6*) were upregulated in involved compared to uninvolved mucosa, *FPR1, OSMR* and *S100A12* were also expressed in normal mucosa, albeit of unrelated individuals.

### Microbiome of aphthous ulcers and Peyer's patches

Ryan *et al*.^22^ reported that the mucosa of patients with IBD trended toward lower microbiota diversity compared to healthy controls, but no individual taxa distinguished inflamed from noninflamed sites within the same individual. The profound induction of pro‐inflammatory cytokines and chemokines (**Cluster 8**) and genes associated with bacterial invasion (**Cluster 4**) in the aphthous ulcers compared to Peyer's patches strongly suggests a response to a pathogen. We used VirusSeeker to examine the 7% of transcript reads that did not map to the human genome. There was no virus in common to all aphthous ulcers or lymph nodes from patients with Crohn's disease. We detected human herpes virus 4 in one Crohn's disease aphthous ulcer, one Crohn's disease lymph node and the mucosa of three Crohn's disease patients as well as one control lymph node (data not shown).

Naftali *et al*.^23^ suggested that *Faecalibacteria* were strongly reduced in the microbiome of patients with ileal Crohn's disease compared with Crohn's disease with colonic involvement. The V3–V4 region of the 16S rRNA gene was amplified from RNA of the six aphthous ulcers that underwent RNA‐seq, an additional six aphthous ulcers from different patients and five of the six Peyer's patches. A similarity of percentages analysis of the 10 most abundant genera in the aphthous ulcers and Peyer's patches microbiomes is provided in Supplementary table 4. Notwithstanding the small group size, consistent with previous literature on the gut microbiome in Crohn's disease,^16^, ^24^ we observed a small depletion in *Faecalibacterium*. In contrast to previous literature, we saw a small depletion of *Escherichia–Shigella* in aphthous ulcers compared to Peyer's patches. As a group, the microbiomes of aphthous ulcers were less diverse than those of Peyer's patches when calculated using various indices (Chao1, *P* = 0.03; Shannon, *P* = 0.03 and Fisher, *P* = 0.03).

## DISCUSSION

IBD is a chronic, debilitating condition that tends to manifest in early adulthood and runs a relapsing and remitting course. Despite a diverse range of monoclonal antibodies targeting specific inflammatory pathways, resistance to therapy is an ongoing clinical problem.^25^ The need for identification of new targets for treatment in the early stages of disease has provided the impetus for generation of numerous gene expression data sets of intact tissue or RNA‐seq of isolated cell populations and more recently the generation of multiple large scRNA‐seq data sets comparing cellular phenotypes in inflamed and healthy intestinal mucosa.^7^, ^8^, ^17^, ^26^, ^27^, ^28^ The analysis of these data sets using dimensionality reduction algorithms, such as UMAP produces a large array of putative subsets of both immune and nonimmune cell types. However, there are two major issues that compromise interpretation. The recovery of resident tissue macrophages by enzymatic disaggregation is substantially lower than their abundance *in situ* and is not necessarily representative. Based upon relative abundance of transcripts encoding known resident macrophage‐specific proteins (CSF1R, C1Q), macrophages contribute about 10–15% of total human intestinal mRNA.^29^ They are readily detected in total tissue mRNA, but the proportion of macrophages detected by scRNA‐seq is much lower.^30^ Second, the isolated macrophages that are profiled in scRNA‐seq data are clearly activated during the process of tissue disaggregation, as has been shown in isolated mouse macrophages from multiple tissues.^31^ Kong *et al*.^8^ identified CCL3^+^CCL4^+^ macrophages as the most abundant population in both normal and inflamed mucosa, but neither *CCL3* nor *CCL4* mRNA is detectable in total RNA‐seq data from normal ileum or colon in the FANTOM5 resource^29^ nor in normal mucosa in our data, whereas *CSF1R* is readily detected. Similarly, Domanska *et al*.^17^ and Elmentaite *et al*.^27^ identified subsets of macrophages in normal human colon that expressed *IL1B*, numerous other pro‐inflammatory chemokines and cytokines, and immediate early genes (e.g. *FOS, JUN*), none of which is detected by RNA‐seq analysis of normal intestinal mucosa.^29^ Against this background, it is difficult to evaluate the predictive value of the GIMATS cellular module proposed by Martin *et al*.^7^ as an indicator of pathogenesis.

As a consequence of random sampling, each intestinal biopsy contains different proportions of structures and cell types enabling deconvolution. Network‐based deconvolution of total RNA‐seq data was used previously to extract signatures of immune cell types in solid tumors. Similarly, the network analysis in Supplementary table 2 highlights co‐expression modules for various intestinal wall cell populations that can provide a framework for interpreting scRNA‐seq data. **Cluster 20** includes markers of several distinct cell types in the intestinal crypts (*ASIC2, CHGA/B, DEFA/B, GCG, LGR5, REG3A, RFX6* and *SOX4*). Because crypts are well‐defined structures, the individual cell types within them do not cluster separately. Conversely, monocyte and macrophage subpopulations vary in relative abundance in different locations within the intestinal wall. Two modules are consistent to some extent with resident lamina propria macrophages (**Cluster 21**) and submucosal macrophages (**Cluster 25**) identified by others^17^ and with data from mouse.^31^ **Cluster 16** contains *CD14*; the key monocytic transcription factor, *CEBPB*; and multiple monocyte‐specific genes.^19^ **Cluster 8** is composed of transcripts that share overexpression in all inflamed tissues, including draining lymph nodes, contains numerous inducible inflammatory genes associated with recruited monocytes.

The extensive published RNA‐seq data on inflamed gut mucosa is limited in that it does not focus on aphthous ulcers, the earliest pathological lesion. Table 1 highlights the distinct classes of transcripts that were increased in aphthous ulcers relative to Peyer's patches or mucosa. The induction of immunoglobulin genes alongside an increase in B‐cell markers (e.g. *CD19*, *CD79*, *BTK*) indicates germinal center formation^32^ and plasma cell differentiation to produce both IgA and IgG. This process may be driven by the upregulation of key chemokine genes (*CCL19*, *CCL21* and *CXCL13*), their receptors (*CCR7* and *CXCR4*) and *TNFSF13B*, the transcript encoding B‐cell activating factor.

Increased levels of transcripts for monocyte‐specific genes (e.g. *CD14, NOD2, S100A8/A9*; **Clusters 8** and **14**) indicate specific infiltration of these cells into the aphthous ulcers. The S100A8/A9 complex, also known as calprotectin, is detectable in stools as well as serum of patients with IBD and has been used as a noninvasive biomarker of disease, likely associated with neutrophils in the intestinal lumen.^33^ Zollner *et al*.^34^ showed that fecal calprotectin was well correlated with another neutrophil‐associated protein, lipocalin 2 (LCN2), that was also increased in aphthous ulcers (Table 1). Immunolocalisation revealed increases in both proteins in neutrophils and monocyte‐macrophages in inflamed mucosa. *LCN2* was also induced in epithelial cells,^34^ and in our RNA‐seq data are about 5–10‐fold more highly expressed than S100A8/A9.

Monocyte recruitment may be linked to increased expression of multiple receptors implicated in transendothelial migration, including selectins (*SELE*, *SELP*, *SELL*), *PECAM1* and *ICAM1*.^35^ Associated with this infiltration, we see expression of transcripts encoding inducible cytokines such as IL1B that are massively more inducible in monocytes than in monocyte‐derived macrophages.^19^ This observation is consistent with our proposal that intestinal inflammation is triggered by hyperresponsiveness of monocytes to intestinal flora. The inducible response may also depend upon priming with interferon−γ (IFNG). **Cluster 27**, also overexpressed in aphthous ulcers, groups *IFNG* with multiple known IFNG target genes, notably *CXCL9/10/11, IL12* and *STAT1*.^36^ Anti‐IFNG antibody (fontolizumab) has been tested clinically in Crohn's patients with some evidence of efficacy.^37^ Interestingly, *ACOD1* is induced in human monocytes and macrophages *in vitro* and functions to divert the citric acid cycle to produce itaconate.^19^ It has been highlighted as an IFNG target and potentially involved in IBD.^38^ However, *ACOD1* mRNA was barely detectable in aphthous ulcers or inflamed mucosa (max 18 TPM).

Our focus on aphthous ulcers also highlights the response of epithelial cells; transcripts associated with the stress response, **Cluster 4** and more specifically **Cluster 14**, were both strongly enriched in aphthous ulcers. A key observation herein is the profound increase in expression of *CEACAM5/6/7* mRNA in aphthous ulcers (Table 1). The encoded proteins are induced by bacterial toxins and provide the major portal for invasion by enteropathogenic *E. coli*.^39^ Other highly induced genes are part of an antimicrobial defense also increased in IBD, including various mucins and the three trefoil factors, that contribute to nonspecific barrier function. However, MUC1, encoded by the most highly induced mucin gene in aphthous ulcers, was shown also to provide an inducible portal for bacterial uptake.^40^ Increased expression of *REG1A*, *LCN2* and *DUOX2* in inflamed mucosa was reported previously.^41^ DUOX2 generates hydrogen peroxide as an inducible defense against microorganisms. Interestingly, biallelic mutation of DUOX2 was recently described in a very early onset IBD patient.^42^ By contrast to rodents, in humans NOS2 (inducible nitric oxide synthase) is not expressed by macrophages.^43^ Conversely, *NOS2* mRNA and nitric oxide production is induced directly in human epithelial cells by invasive *E. coli*, *Salmonella* and *Shigella* species.^44^ Increased expression of IDO1 in epithelial cells has also been reported in ilea of Crohn's disease patients.^45^

**Figure 4:**
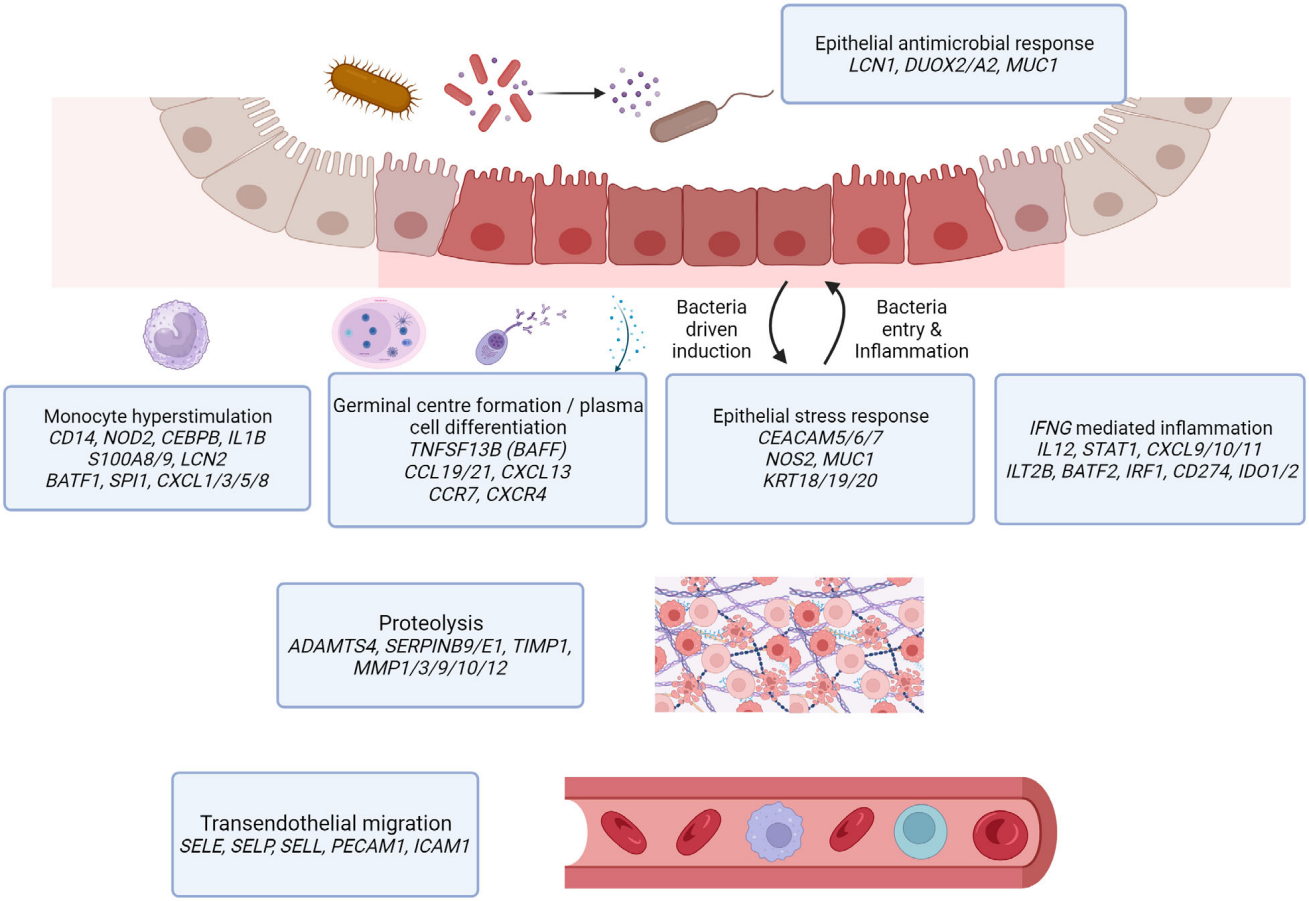
Inflammatory response pathways informed by RNA‐seq cluster analysis of aphthous ulcers early in Crohn's disease inflammation. At the mucosa and lamina propria, highly expressed genes formed distinct functional groups suggestive of bacterial antigen‐induced epithelial stress, monocyte stimulation, IFNG priming and plasma cell differentiation along with germinal center formation. Distinctly upregulated subsets of genes were observed for endothelial cell migration and proteolysis functions.

Taken together, both the increased expression of inflammatory cytokines by recruited monocytes and associated alterations in epithelial gene expression in aphthous ulcers strongly implicate a response to a microbial stimulus (a diagrammatic overview of these changes is presented in Figure 4). In contrast, the absence of significant epithelial cell injury in the mucosa adjacent to aphthous ulcers (Figure 2) argues against a global dysbiosis causing a breakdown in epithelial integrity as the initiating factor in the development of Crohn's disease arising from aphthous ulcers.

The analysis herein and previous studies^16^, ^22^ provide very limited evidence for the selective enrichment or depletion of specific bacterial taxa in aphthous ulcers. This does not preclude a possible role for a microorganism as the initial trigger of focal inflammation in Peyer's patches. Subsequent chronic inflammation may lead to disease‐associated dysbiosis, but the evidence for this has been questioned in a recent systematic review of the published studies.^46^

Our analysis of a relatively small data set demonstrates that it is possible to extract information that complements, and in some cases clarifies, data generated by scRNA‐seq. The results are consistent with the concept that Crohn's disease pathology is triggered by a microbial stimulus at the site of luminal antigen sampling. Genetic susceptibility may determine the nature of the host immune response to a specific “cognate” microbial trigger (which may differ between individuals) and/or regulate the nature, magnitude and duration of the response in those who progress to develop the full clinical manifestations of Crohn's disease.

## METHODS

### Human subjects

The study was approved by the ACT Health Human Research Ethics Committee (ETH.5.07.464) and the Australian National University Human Ethics Committee (2012/596) and written informed consent was obtained from all subjects. Crohn's disease diagnoses were based on typical clinical presentation, and endoscopic and histological findings. Biopsies used for the analysis were obtained from the terminal ileum only, for both patients and controls, unless otherwise stated. Biopsies were also sent for histological assessment in parallel to confirm the endoscopic findings. One operator obtained the biopsies from the six Peyer's patch samples and six aphthous ulcer samples used in the direct comparisons. We did not confirm the classification of biopsies (Peyer's patches or aphthous ulcers) on the individual biopsies used for RNA extraction, but separate biopsies did correspond to the endoscopic impression at histology. Lymph nodes were obtained from surgical resections.^47^ Excluding patients who underwent surgical resection, patients were not on pharmacological treatment for Crohn's disease, antibiotics or nonsteroidal anti‐inflammatory medications. Details of the patients are provided in Supplementary table 5a, b.

### RNA extraction and NextSeq 500 sequencing

RNA was extracted from (1) biopsies of aphthous ulcers and adjacent endoscopically unaffected mucosa in Crohn's disease patients, and Peyer's patches and adjacent mucosa in controls; (2) biopsies of established inflammation comprising active ulceration and adjacent endoscopically unaffected mucosa in Crohn's disease patients and healthy mucosa from non‐IBD subjects and (3) resected specimens of ulcerated mucosa, adjacent unaffected mucosa and lymph nodes from Crohn's disease patients, and resected mucosa and lymph node specimens from non‐IBD subjects.

RNA was extracted using Qiagen RNeasy Mini kits (Qiagen, Aarhus, Denmark). Each biopsy was mechanically homogenized using a Qiagen TissueLyser II, and an oncolumn DNase digestion step was performed as per the kit protocol. An aliquot of denatured RNA was quantified using an Agilent 2100 Bioanalyzer with RNA 6000 Nano LabChips (v2). NextSeq 500 libraries were prepared at the Biological Research Facility (Australian National University) using 1 μg RNA for each sample. Truseq‐stranded RNA LT kits and NextSeq 500/550 High output kits (Illumina, San Diego, CA, USA), in a 150 × 150 bp (paired‐end) format, were used as per the manufacturer's instructions. Whole‐genome transcripts were assessed for quality using FASTQC, trimmed using Trimmomatic and aligned to the human reference genome (GRCh38) using subread mapper. Fragment counts were obtained using featureCount, and expression values were normalized using the trimmed mean of *M* values normalization method (TMM).^48^ Reads that did not map to the human genome in RNA‐seq data from each sampled tissue were used to mine virus sequences using the VirusSeeker pipeline.^49^

For qRT‐PCR validation of gene expression differences, RNA was extracted from RNAlater®‐stabilized frozen biopsies or approximately 30 mg of resected bowel tissue, using RNeasy Mini Kits (Qiagen). Samples were treated for DNA contamination using RNase‐Free DNase (Qiagen). cDNA was synthesized using 500 ng total RNA and Superscript III cDNA First Strand Synthesis kit (Invitrogen, Mt Waverley, Australia) with random primers. Quantitative PCR was carried out using a Quantstudio 12 K system, SYBR Green (Invitrogen, Carlsbad, CA, USA). The primers are listed in Supplementary table 6. PCR reactions were conducted under the following conditions: 95°C for 3 min, followed by 45 cycles of 95°C for 10 s, 60°C for 20 s and 72°C for 20 s; one cycle of 95°C for 1 min and 60°C for 1 min and 71 cycles from 60°C to 95°C for melt curve analysis with 0.5°C increases. Two experiments were run in duplicate and analyzed using *GAPDH* and *ACTB* as reference genes. Taqman™ fast advanced protocol (Applied Biosystems, Foster City, CA, USA) was used for *NOD2* (Hs01550753_m1) and *LILRA2* (Hs01597933_g1). All experiments were carried out using two biological and three experimental replicates.

### Data analysis

#### Transcriptional network analysis

Transcripts that were not detected in at least six samples were removed to minimize stochastic sampling errors. Network analysis was performed using the program, BioLayout (http://biolayout.org) or a further development of this platform, Graphia (https://graphia.app/).^50^ By contrast to the widely used WGCNA approach,^51^ this network method generates an all *versus* all correlation matrix to which it applies a correlation threshold cut‐off, removing outliers. This thresholded matrix is used to generate a true correlation graph from which co‐expression modules are then defined using the MCL algorithm, which further refines and removes outliers. The outcomes are similar to WGCNA, but modules defined by this approach are biologically enriched over those defined by WGCNA.^50^ Pairwise Pearson correlations (*r*) were calculated between samples to produce a sample‐to‐sample correlation matrix and inversely between all pairs of genes to produce a gene‐to‐gene correlation matrix. Gene co‐expression networks were generated from the matrix, in which nodes represent genes and edges represent correlations between nodes above a defined correlation threshold. An *r* value threshold of 0.75 was used for all gene‐to‐gene analyses unless otherwise stated. Further analysis used the Markov algorithm (MCL) with an inflation value of 2.0 to identify groups of highly connected genes within the overall topology of the network.

Differential gene expression analyses were also performed using generalized linear models in edgeR,^48^ with a false discovery rate (FDR) applied after correcting for multiple hypothesis testing using the Benjamini and Hochberg method.^52^

Gene ontology (GO) terms were derived from the Gene Ontology Resource (http://geneontology.org) and the enrichment of GO terms was assessed using the PANTHER overrepresentation test (PANTHER).

#### Microbiome analysis

The V3–V4 region of the 16S rRNA gene was amplified from RNA derived from tissue biopsies, and the corresponding amplicon library was sequenced on an Illumina MiSeq platform. Qiime2‐2019.10 was used for the generation of alpha and beta diversity metrics, distance analyses and for visualizing the data.

## AUTHOR CONTRIBUTIONS

**Phillip J Whiley:** Data curation; formal analysis; investigation; project administration; validation; writing – original draft; writing – review and editing. **Mukta Das Gupta:** Formal analysis; investigation; writing – review and editing. **Ojas VA Dixit:** Data curation; investigation. **Hardip Patel:** Data curation; formal analysis; investigation. **Guoyan Zhao:** Formal analysis; investigation. **Susan J Connor:** Data curation; investigation. **Kim M Summers:** Data curation; validation; writing – review and editing. **David A Hume:** Conceptualization; data curation; formal analysis; funding acquisition; investigation; methodology; validation; visualization; writing – original draft; writing – review and editing. **Paul Pavli:** Conceptualization; data curation; formal analysis; funding acquisition; investigation; methodology; project administration; resources; supervision; validation; visualization; writing – original draft; writing – review and editing. **Claire L O'Brien:** Conceptualization; data curation; formal analysis; funding acquisition; investigation; methodology; project administration; resources; supervision; validation; visualization; writing – original draft; writing – review and editing.

## CONFLICT OF INTEREST

The authors have no conflicts of interest to declare.

## Supporting information


Supplementary figures 1 and 2

Supplementary tables 1‐6

## Data Availability

The data that support the findings of this study are available upon request from the corresponding author. The data are not publicly available due to privacy or ethical restrictions.
