# Supplementary material for: Transcriptomic signatures of host immune responses in aphthous ulcers, the earliest lesions of Crohn's disease, suggest that bacterial uptake, rather than global dysbiosis, is the initiating factor
Source: Immunol Cell Biol. 2025 May 19;103(5):473–84. doi: 10.1111/imcb.70031 (PMC12108696; doi:10.1111/imcb.70031)
Supplement: Supplementary file 1 — Supplementary figures 1 and 2 [file IMCB-103-473-s002.docx]

Supplementary Figures


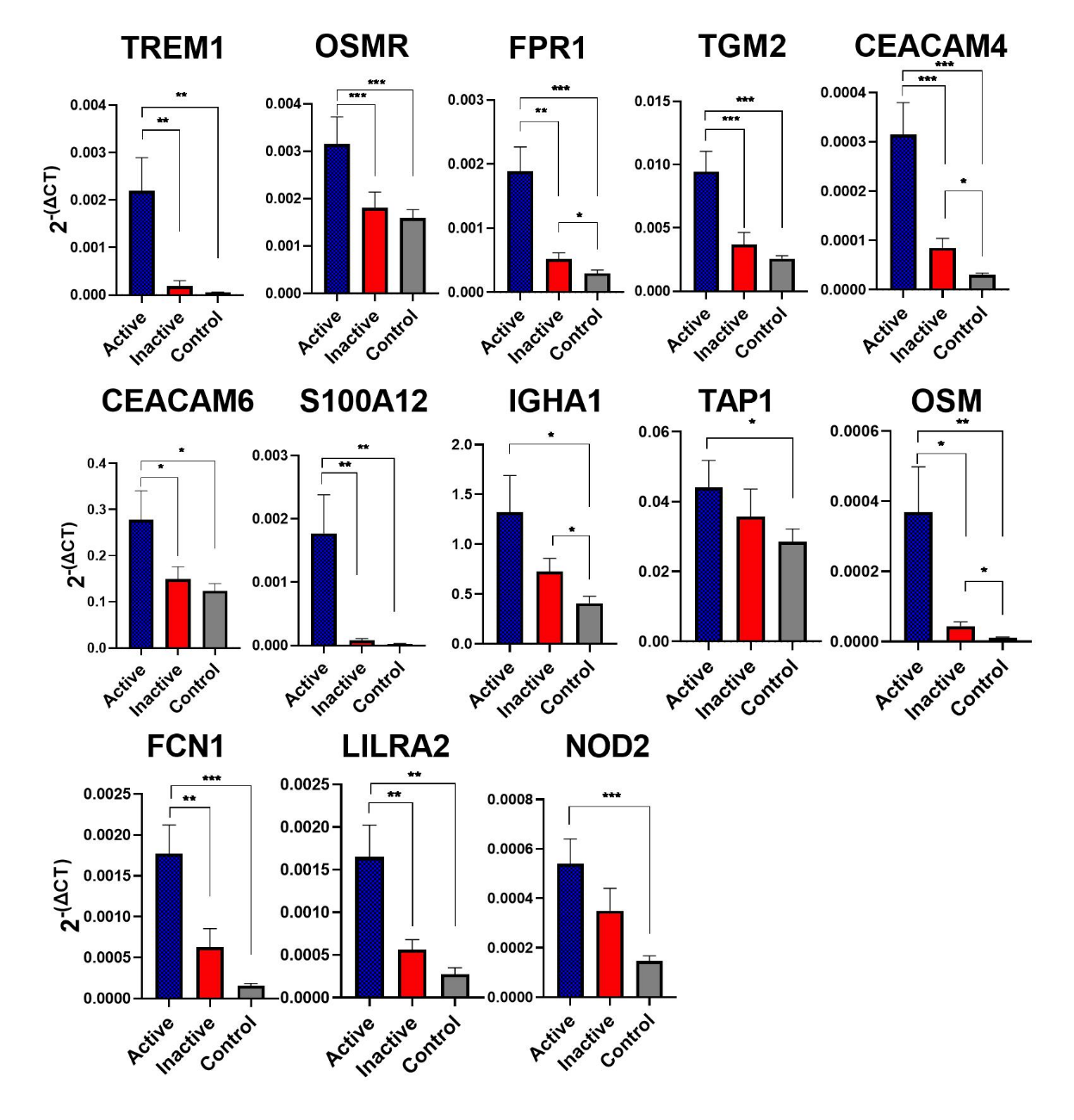


**Supplementary figure 1.** Quantitative Real-time PCR comparing actively inflamed mucosa from the terminal ileum against adjacent from the same individuals with respect to the control group. Apart from TAP1, expression of genes in the active mucosa was elevated in the panel of genes selected to exemplify key functional roles.


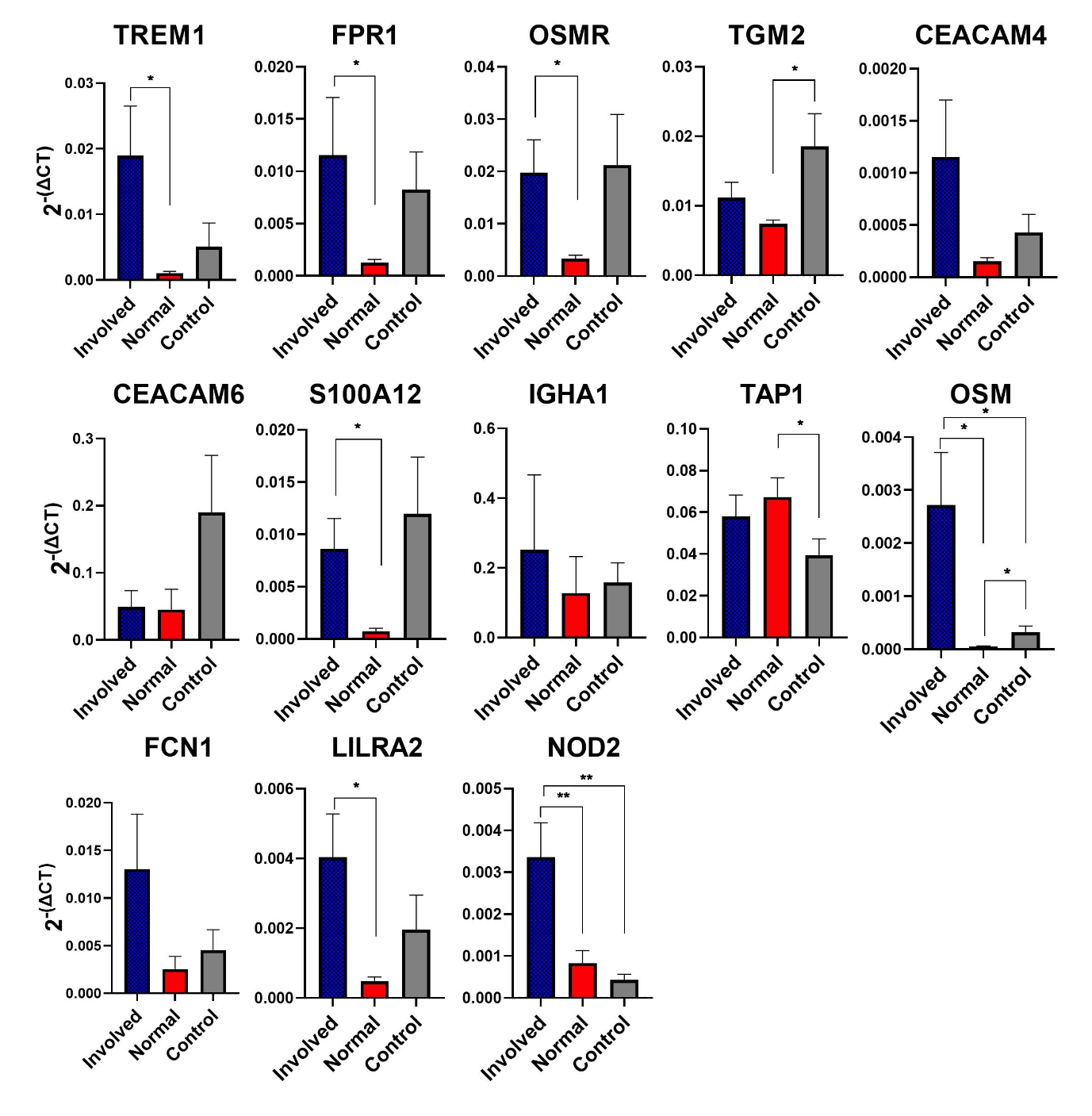


**Supplementary figure 2.** Quantitative Real-time PCR comparing actively inflamed mucosa from the colon against adjacent from the same individuals with respect to the control group. In the colon, expression the genes from the selected panel in inflamed and involved mucosa was less pronounced compared to the terminal ileum in Supplementary figure 1.
